# Dangerous Relations in the Arctic Marine Food Web: Interactions between Toxin Producing *Pseudo-nitzschia* Diatoms and *Calanus* Copepodites

**DOI:** 10.3390/md13063809

**Published:** 2015-06-16

**Authors:** Sara Harðardóttir, Marina Pančić, Anna Tammilehto, Bernd Krock, Eva Friis Møller, Torkel Gissel Nielsen, Nina Lundholm

**Affiliations:** 1 Natural History Museum of Denmark, University of Copenhagen, Sølvgade 83S, 1307 Copenhagen, Denmark; E-Mails: marina.pancic@snm.ku.dk (M.P.); atammilehto@snm.ku.dk (A.T.); nlundholm@snm.ku.dk (N.L.); 2 National Institute of Aquatic Resources, Technical University of Denmark, Charlottenlund Slot, Jægersborg Allé 1, 2920 Charlottenlund, Denmark; E-Mail: tgin@aqua.dtu.dk; 3 Alfred-Wegener-Institut für Polar- und Meeresforschung, Ökologische Chemie, Am Handelshafen 12, 27570 Bremerhaven, Germany; E-Mail: bernd.krock@awi.de; 4 Arctic Research Center, Department of Bioscience, Roskilde, Aarhus University, Frederiksborgvej 399, P.O. Box 358, 4000 Roskilde, Denmark; E-Mail: efm@bios.au.dk

**Keywords:** *Calanus* copepodites, *Pseudo-nitzschia seriata*, *P. obtusa*, grazing, induction, chemical ecology, toxin production, domoic acid

## Abstract

Diatoms of the genus *Pseudo-nitzschia* produce domoic acid (DA), a toxin that is vectored in the marine food web, thus causing serious problems for marine organisms and humans. In spite of this, knowledge of interactions between grazing zooplankton and diatoms is restricted. In this study, we examined the interactions between *Calanus* copepodites and toxin producing *Pseudo-nitzschia*. The copepodites were fed with different concentrations of toxic *P. seriata* and a strain of *P. obtusa* that previously was tested to be non-toxic. The ingestion rates did not differ among the diets (*P. seriata*, *P. obtusa*, a mixture of both species), and they accumulated 6%–16% of ingested DA (up to 420 µg per dry weight copepodite). When *P. seriata* was exposed to the copepodites, either through physical contact with the grazers or separated by a membrane, the toxicity of *P. seriata* increased (up to 3300%) suggesting the response to be chemically mediated. The induced response was also triggered when copepodites grazed on another diatom, supporting the hypothesis that the cues originate from the copepodite. Neither pH nor nutrient concentrations explained the induced DA production. Unexpectedly, *P. obtusa* also produced DA when exposed to grazing copepodites, thus representing the second reported toxic polar diatom.

## 1. Introduction

The marine biotoxin domoic acid (DA) causes amnesic shellfish poisoning (ASP) in humans [1,2], and exposure to DA is known to have harmful effects on animals in the marine food web, e.g., sea birds and mammals [3,4]. DA is produced by species of the diatom genera *Nitzschia* and *Pseudo-nitzschia* as a secondary metabolite [5,6]. DA accumulates in marine organisms that feed on phytoplankton, e.g., planktivorous fish (such as sardines), bivalves and copepods [7,8,9], which may then serve as vectors for DA in the food web. Despite the grim effects that DA-producing diatoms have on higher trophic levels, only a few studies have explored the relations between toxic diatoms and their grazers [10,11].

*Pseudo-nitzschia* is a globally distributed diatom genus, of which many species form extensive blooms. Fourteen of the 39 described species are known to be toxigenic [11]. The first recorded ASP incident in 1987 in Prince Edward Island, Canada [1], resulted in increased research interest, and surveillance programs now monitor concentrations of *Pseudo-nitzschia* and/or levels of DA in mollusks. These research and monitoring efforts have particularly expanded our knowledge of the ecology, distribution, taxonomy, and toxin-production of *Pseudo-nitzschia* in temperate, subtropical and tropical areas. The polar regions, however, have received much less attention. No records of a toxigenic *Pseudo-nitzschia* species exist from the Antarctic and the first, and so far only, record of a toxin-producing diatom in the Arctic is *P.* *seriata* [12,13].

Several species of copepods are known to graze on toxic *Pseudo-nitzschia.* Most studies have not detected any reduction in grazing on toxic *versus* non-toxic *Pseudo-nitzschia* [9,14,15,16,17]. The only study conducted on arctic copepods, *Calanus* spp., did not find significant differences in the overall weight-specific ingestion of toxic and non-toxic species in three *Calanus* species. However, discontinuous grazing rates were detected, indicating that two of the species, *C. finmarchicus* and *C. hyperboreus*, were temporally affected when fed with toxic *P. seriata*. This suggests that DA may act as a grazing deterrent against copepods [18]. A similar impact was seen on the grazing pattern in the krill *Euphausia pacifica*, when grazing on toxic *P. multiseries* was compared to non-toxic *P. pungens* [19].

*Pseudo-nitzchia seriata* increased production of DA when exposed to grazing adult *Calanus* copepods [20], an effect which may be related to defense against grazing, and the response was found to be chemically mediated. Changes in nutrients (silicate, nitrate, ammonium and phosphate), and changes in pH levels are known triggers for DA production in *Pseudo-nitzschia* species [21,22,23]. However, the few studies that have measured changes in nutrients when investigating induced responses in phytoplankton by zooplankton, have not found the nutrients to be the inductive factor [24,25,26,27].

In the Arctic, copepods of the genus *Calanus* dominate the mesozooplankton. Before the phytoplankton spring bloom, the copepods ascend from the depth to feed, reproduce and spawn [28]. After spawning, the adult *Calanus* copepods descend to the water near the bottom for hibernation [29]. Thereafter, the younger stages are among the most abundant mesozooplankton and are often key grazers during the post bloom period [30,31]. The grazing studies mentioned previously were all performed on adult females. It is not known if the younger stages graze on *Pseudo-nitzschia* and if they do, whether they are more vulnerable to the toxins. To our knowledge, no other studies have explored the effect of DA on younger stages of copepods and whether they retain DA, neither have their effect on toxin production in phytoplankton been explored.

The aims of the present study were to investigate the interaction between *Calanus* copepodites and *Pseudo-nitzschia*, *i.e.*, (1) if copepodites select between *Pseudo-nitzschia* species of different toxicity or size; (2) if grazing on toxic *Pseudo-nitzschia* affects grazing rates and/or mortality of the copepodites; (3) if copepodites retain DA; (4) if grazing pressure from the copepodites induces DA production in a toxic (*P. seriata*) and a non-toxic (*P. obtusa*) *Pseudo-nitzschia* species; and (5) if the induced DA production is mediated because of changes in the major inorganic nutrients or pH, or due to waterborne chemical cues from the copepodites.

## 2. Results

### 2.1. Temporal Grazing Experiment

#### 2.1.1. Toxicity

At the beginning of the experiment, the copepodites and the *P. obtusa* cells did not contain DA, whereas *P. seriata* cells contained low amounts of DA, ~0.1 pg cell^−1^ (level of detectio *n =* 0.003 pg DA cell^−1^) (Table 1). Domoic acid cell quotas increased significantly in both *P. seriata* and *P. obtusa* when exposed to grazers, but not in the control without copepodites (Table 1). This is the first report of DA production by *P. obtusa*. *P. seriata* produced more DA than *P. obtusa* per cell (*t*-test, *P* < 0.001) (Table 1), and also per volume, *i.e.*, considering the larger biovolume of *P. seriata* (~2.2 × 10^−3^ and ~0.5 × 10^−3^ pg DA per µm^3^, for *P. seriata* and *P. obtusa*, respectively) (*t*-test, *P* = 0.003). Dissolved DA was not measured. At the end of the experiment, the copepodites retained 0.1 ± 0.0 ng DA µg C^−1^ after grazing on *P. obtusa*, the *P. obtusa* cells produced 0.4 ± 0.1 × 10^−4^ ng DA µg C^−1^. After grazing on *P. seriata* for 39 h, the copepodites retained 0.6 ± 0.2 ng DA µg C^−1^, the *P. seriata* cells produced 1.9 ± 4.4 × 10^−4^ ng DA µg C^−1^. DA measured in the copepodites ranged from 8.2 to 69.7 ng DA per individual, with the lowest amount after grazing on *P. obtusa* and the highest after grazing on *P. seriata* (Mann-Whitney Rank Sum Test, *P* = 0.03) (Table 1). Of the ingested DA after grazing on *P. obtusa*, the copepodites retained 6%, and 13%–16% when grazing on *P. seriata* and mixture of both, respectively.

**Table 1:** Grazing experiment. *Pseudo-nitzschia* cell density (cells mL^−1^) and domoic acid (DA) cell quota (pg DA cell^−1^) in *P. seriata* and *P. obtusa* in treatments and controls. *Calanus* total ingested (ng DA cop^−1^) and retained DA per copepodite, given in measured DA (ng DA cop^−1^), by dry weight (µg DA g DW cop^−1^) and as percent of ingested DA. Results are given as * = significant difference from start to end, and ** for significant difference between control and treatment. LOD = level of detection. (LOD for *P. obtusa* was 0.001 pg DA cell^−1^).

	*Pseudo-nitzschia*				*Calanus*			
	Number of Cells		DA Cell Quota		Ingested DA	Retained DA		
	(Cells mL^−1^)		(pg DA Cell^−1^)		(ng DA cop^−1^)	(ng DA cop^−1^)	(µg DA g DW cop^−1^)	% of Ingested DA
	Initial	End	Initial	End				
Controls								
*P. seriata*	3964 ± 40	5048 ± 102	0.1 ± 0.0	0.1 ± 0.0				
*P. obtusa*	5061 ± 32	8907 ± 683	<LOD	<LOD				
*P. seriata + P. obtusa*	7144 ± 103	8617 ± 122	0.1 ± 0.0	0.2 ± 0.0				
Treatments								
*P. seriata* + copepodites	3951 ± 52	1901 ± 187	0.1 ± 0.0	2.6 ± 0.6 *^,^ **	530 ± 21	70 ± 18	410 ± 112	13 ± 4
*P. obtusa* + copepodites	5951 ± 285	4988 ± 570	<LOD	0.2 ± 0.1 *^,^ **	137 ± 37	8 ± 2	48 ± 10	6 ± 2
*P. seriata + P. obtusa +* copepodites	7033 ± 97	4262 ± 249	0.1 ± 0.0	3.7 ± 0.6 *^,^ **	275 ± 70	42 ± 8	246 ± 51	16 ± 4

#### 2.1.2. Grazing and Fecal Pellet Production

**Figure 1:**
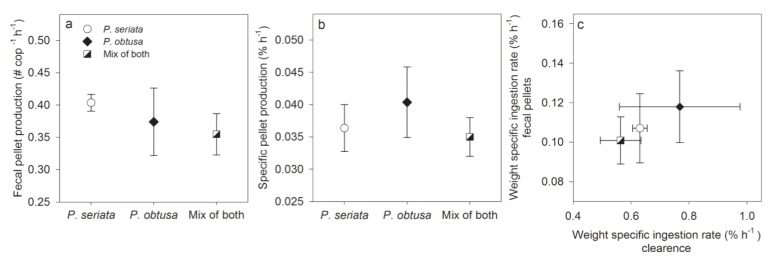
Fecal pellet production and weight-specific ingestion rate in the 39 h grazing experiment. (**a**) Fecal pellet production (cop^−1^ h^−1^); and (**b**) specific fecal pellet production (SPP) (% h^−1^) when grazing on *P. seriata*, *P. obtusa* or a mixture of both species. No significant differences were found among treatments; (**c**) Weight-specific ingestion rate (% h^−1^), calculated from fecal pellet production and from clearance were plotted against each other for the three diets. The weight-specific ingestion rate was statistically higher when calculated from clearance, than calculated from fecal pellet egestion.

**Figure 2:**
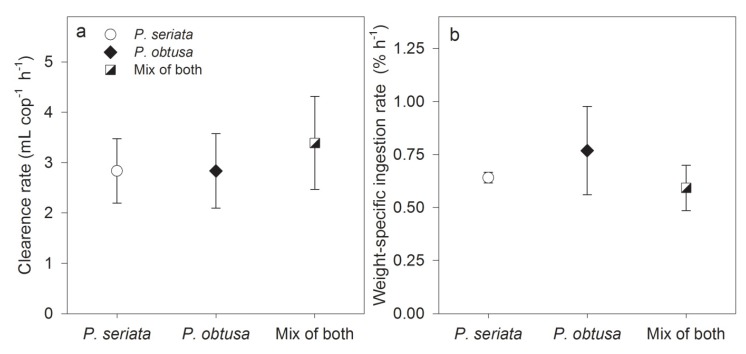
Grazing in the 39 h experiment. (**a**) Mean clearance rate (mL^−1^cop^−1^ h^−1^, mean ± SD) for grazing on *P. seriata*, *P. obtusa* or a mixture of both species; (**b**) Weight-specific ingestion rates (% h^−1^, mean ± SD) for grazing on *P. seriata*, *P. obtusa* or a mixture of both species. No statistical differences were found among treatments.

The copepodites produced fecal pellets on all diets (Figure 1a) proving that the copepodites grazed on the toxic algae. Of the 146 copepodites used in the experiment, 138 were alive and vital at the end of the experiment, and no dead copepodites were found in the samples at the end. Two of the missing copepodites were discovered in the cell enumeration samples, six were missing. The mean clearance rate for the 39 h was the same (2.8 ± 0.7–3.3 ± 0.6 mL cop ^−1^ h^−1^) for all three treatments (one-way ANOVA, *F*_2,9_ = 0.3, *P* = 0.8) (Figure 2a); and there was not signifcant difference among ingestion rates, either when grazing on *P. seriata*, *P. obtusa* or a mixture of both species (one-way ANOVA, *F*_2,9_ = 0.3, *P* = 0.7). We found no significant changes in weight-specific ingestion rates between the light and the dark periods in any of the diets (RM ANOVA, *P* > 0.05) (Supplementary Figure S1), and the average ingestion rate during the 39 h (0.56% ± 0.1%–0.77% ± 0.2% h^−1^) did not differ among the diets (one-way ANOVA, *F*_2,9_ = 0.3, *P* = 0.8) (Figure 2B).

Fecal pellet production was highest when grazing on *P. seriata,* and specific fecal pellet production (SPP) was highest when grazing on *P. obtusa* (Figure 1b). However, no significant differences were found in number of fecal pellets produced (one-way ANOVA, *F*_2,9_ = 1.3, *P* = 0.4) nor in SPP among the diets (one-way ANOVA, *F*_2,9_ = 1.0, *P* = 0.4). Comparison of the weight-specific ingestion rates, based on clearance of carbon or based on fecal pellet egestion, showed a higher ingestion rate when based on clearance, than egestion when grazing on all three diets (*t*-tests; *P. seriata* and mix of both species *P* < 0.001, and *P. obtusa* Mann-Whitney Rank Sum Test, *P* = 0.03). For both calculation methods, a comparison of the ingestion rate among the diets showed the highest ingestion rate on *P. obtusa*, and the lowest on the mixed diet (Figure 1c).

#### 2.1.3. Growth Conditions

The initial cell density in each of the diet treatments was the same as in the controls (*t*-test; for *P. seriata* *P* = 0.7; *P. obtusa P* = 0.1 and in the treatment with mixed cultures: *P. seriata* *P* = 0.9 and *P. obtusa* *P* = 0.1). Overall, no significant changes or trends in changes of concentrations of phosphate (PO_4_^3−^), ammonium (NH_4_^+^) and nitrate (NO_3_^−^) were observed, although single samples differed either from start to end or between control and treatment (see details in Supplementary Table S1). Silicate (Si(OH)_4_) concentrations decreased significantly in all the controls from start to end of the experiment, whereas in the treatments with copepodites there was no significant change. The pH levels were 8.1 ± 0.1 in the controls and the treatments at the start of the experiment and 8.1 ± 0.03 at the end of the experiment, and no overall significant changes were observed among the treatments containing copepodites and the controls (see details in Supplementary Table S1).

### 2.2. Induction Experiments

#### 2.2.1. Toxicity

**Figure 3:**
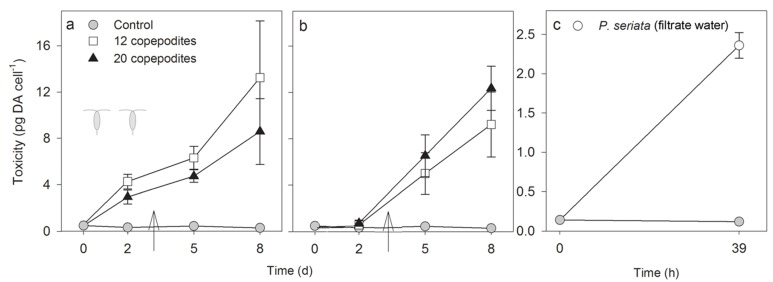
Domoic acid cell quota (pg DA cell^−1^, mean ± SD) of *P. seriata* when grazed by two different concentrations of copepodites and in the control. (**a**) Flask A with copepodites and *P. seriata* (*n =* 4); (**b**) Flask B with only *P. seriata* cells separated from the copepodites by a 2 µm membrane (*n =* 4). The control contains *P. seriata* cells (*n =* 3). The arrows indicate the time when cells were added to ensure the copepodites had enough food; (**c**) Toxicity of *P. seriata* (pg DA cell^−1^, mean ± SD) when grown in the filtrate water where copepodites had been grazing on another diatom species (*Thalassiosira* sp.) Note the different scales in (**c**).

Domoic acid cell quota (toxicity) in *P. seriata* increased significantly in both copepodite density treatments, both in flask A with cells in direct contact with the grazers (Figure 3a), and in flask B with cells separated from the grazers (Figure 3b) (RM ANOVA, *P-values <* 0.05, except for flask A day 2 to 5, where non-induced cells were added on day 3). Overall, the DA cell quota gradually increased in flask A from day 0 to day 8, whereas the response was delayed in flask B (Figure 3). In the control, DA cell quota did not change during the experiment, it was consistently in the range 0.3 ± 0.1 to 0.4 ± 0.0 pg DA cell^−1^ (RM ANOVA, *F*_2,6_ = 38.8, *P* = 0.08). From day 0 to 2, toxicity of *P. seriata* increased significantly only in flask A (*t*-tests, with 12 copepodites *P* < 0.001 and with 20 *P* = 0.003). On day 2, the DA cell quota was significantly higher in flask A than in B (*t*-tests: *P* = 0.002 and *P* < 0.001 for 12 and 20 copepodites, respectively). After day 2, toxin content was the same in flask A and B in both concentrations of copepodites (*t*-tests, *P* > 0.05). The highest level of DA was 13.3 ± 4.9 pg DA cell^−1^ in flask A with 12 copepodites on day 8 (Figure 3 and Supplementary Table S2). In the experiment where cells of *P. seriata* were grown in filtrate water, where the copepodites had previously been grazing on *Thalassiosira* sp., the increase in DA cell quota was from 0.2 ± 0.0 to 2.4 ± 0.2 pg DA cell^−1^ in 39 h (paired *t*-test, *P* = 0.002) (Figure 3c).

#### 2.2.2. Growth Rate, Cell Density and Growth Condition

**Figure 4:**
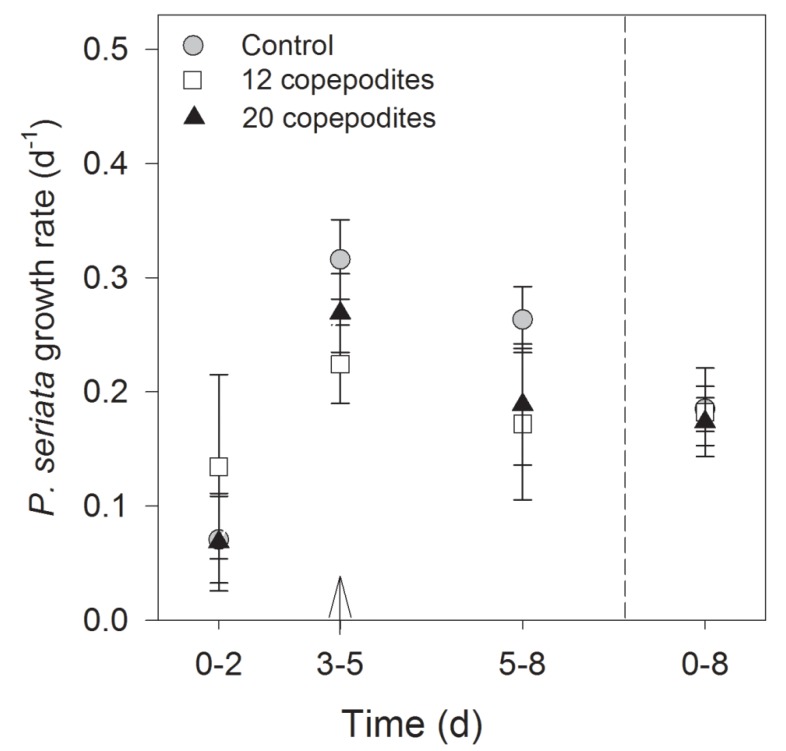
Growth rate (day^−1^) in flask B containing only *P. seriata* cells, and the controls. On day 3, the flasks were diluted with 1/10 L medium (arrow) to compensate for adding cells to the other site of the chamber where the copepodites had reduced the cell concentration drastically. No statistical differences were found among treatments.

**Figure 5:**
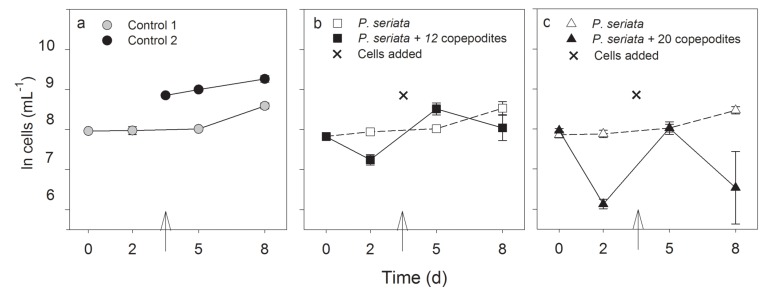
(**a**) Cell concentrations (ln cells mL^−1^, mean ± SD) in the induction experiment. (**a**) The two controls (both *n =* 3); (**b**) Cell concentrations in flask A with 12 copepodites and flask B with only *P. seriata* cells separated from flask A by a 2 µm membrane (*n =* 4). (**c**) Cell concentrations in flask A with 20 copepodites and flask B with only cells separated from flask A by a 2 µm membrane (*n =* 4). The cross and arrows indicate the time and the amount for *P. seriata* cells or 1/10 L medium addition.

The initial cell concentrations were the same in all treatments and in the controls at the start of the experiment (one-way ANOVA, *F*_2,11_ = 3.7, *P* = 0.05). Mean growth rates (eight days) in all incubators containing only *P. seriata* cells, *i.e.*, flasks B and controls, were the same (Kruskal-Wallis test, *P* = 0.7), indicating similar growth conditions (Figure 4). The growth rates were slightly lower during the first two days, 0.1 ± 0.9 d^−1^ and stabilized thereafter around 0.3 ± 0.1 day^−1^. The cell density was significantly lower in flask A than in flask B on day 2 in both treatments (12 and 20 copepodites) (Figure 5b) (*t*-test, *P* < 0.001 and Mann-Whitney Rank Sum Test, *P* = 0.029) illustrating that the copepodites grazed on the toxic cells. Despite the addition of cells in flask A on day 3, the cell numbers were significantly lower in flask A than in flask B on day 8 in both treatments (Figure 5) (*t*-test, *P* = 0.018, and Mann-Whitney Rank Sum Test, *P* = 0.029).

Overall, the nutrient measurements showed a significantly larger decrease in silicate concentration in the control than in the treatments, relatively stable concentrations of phosphate and ammonium throughout the experiment (although single samples differed either from start to end or between control and treatment (see details in Table 2)), and a significantly higher end concentration of nitrate in the treatments than in the control (Table 2). Because only two measurements were available for the initial concentration of nitrate, it was not included in the statistical tests. The initial NO_3_:Si(OH)_4_:PO_4_^3−^ ratios were ~23:1:1. The pH levels were 8.1 at the start of the experiment and varied between 8.1 and 8.2 at the end of the experiment (Table 2), with no statistical difference between the treatments and the controls.

**Table 2:** Induction experiments. Nutrient concentrations and pH levels at the start and the end of the induction experiments. *n* is the number of copepoditesr. Values are given as mean ± SD. Results are given as * = significant difference between start and end and ** = significant difference between the control and the treatment.

Time (day)/(h)	Treatment	Si(OH)_4_ (µmol L^−1^)	PO_4_^3−^ (µmol L^−1^)	NH_4_^+^ (µmol L^−1^)	NO_3_^−^ (µmol L^−1^)	pH
0	Initial	5.7 ± 0.6	5.6 ± 0.6	38.3 ± 1.6	131.6 ± 5.6	8.10
8	Control	0.0 *	3.5 ± 0.3 *	25.6 ± 1.0 *	99.3 ± 6.3	8.12 ± 0.02
8	Flask A, *n =* 12	1.3 ± 1.0 *	6.6 ± 1.2 **	28.8 ± 2.3	182.6 ± 20.7 **	8.16 ± 0.02
8	Flask A, *n =* 20	3.6 ± 0.7 *^,^ **	7.1 ± 0.5 *^,^ **	33.9 ± 0.7 **	191.2 ± 4.2 **	8.12 ± 0.02
8	Flask B, *n =* 12	0.4 ± 0.7 *	5.5 ± 1.1	25.9 ± 1.4	164.2 ± 17.7 **	8.18 ± 0.00
8	Flask B, *n =* 20	0.4 ± 0.3 *	5.1 ± 0.1	27.9 ± 1.5	161.4 ± 10.5 **	8.11 ± 0.01
0	Initial	6.7 ± 0.6	7.0 ± 0.2	38.8 ± 1.7	164.8 ± 12.5	8.06 ± 0.00
39 h	Control	3.6 ± 0.3 *	7.4 ± 0.1 *	39.9 ± 1.0	177.7 ± 1.0	8.09 ± 0.01
39 h	End	3.7 ± 0.31 *	8.0 ± 0.4 *	43.3 ± 0.7 *^,^ **	197.7 ± 1.1 *^,^ **	8.10 ± 0.01

#### 2.2.3. Grazing and Retained Domoic Acid

**Figure 6:**
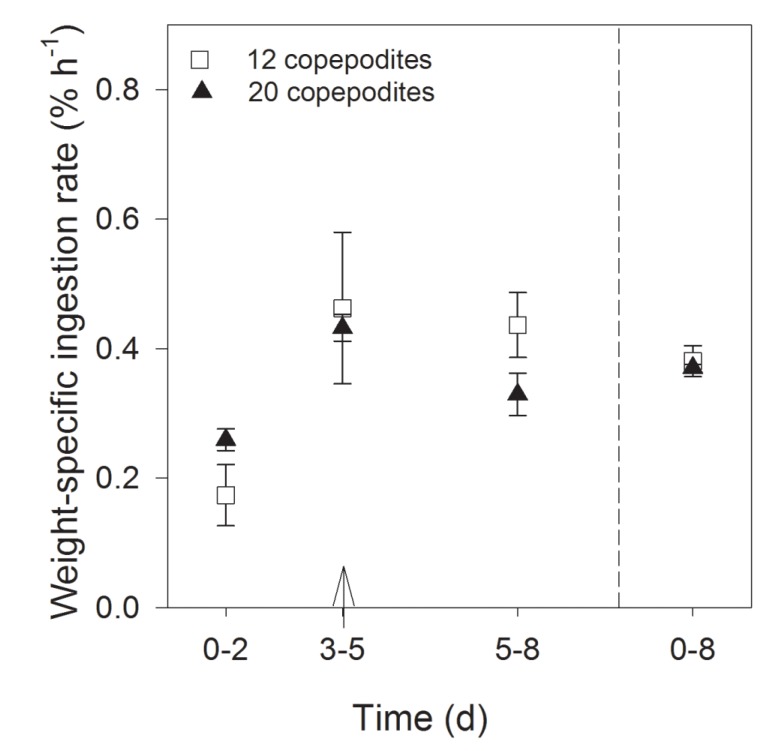
Weight specific ingestion rate of the two concentrations of grazers (% h^−1^, mean ± SD). The arrows indicate the time when cells were added to ensure the copepodites had enough food.

The average clearance rate was 0.7 ± 0.1 mL cop^−1^ h^−1^ in flask A with 12 copepodites and 1.1 ± 0.2 mL cop^−1^ h^−1^ with 20 copepodites. The average weight specific ingestion rate (0–8 days) was the same in both treatments (0.4% ± 0.0% h^−1^). The lowest ingestion rate was measured between days 0–2 and highest between days 3–5 after additional cells had been added (Figure 6). At the end of the experiment, 106 of the initial 128 copepodites were alive and vital. In the treatment with 12 copepodites, one was dead, and in the treatment with 20 copepodites, six copepodites were dead, the remaining copepodites were not found. After eight days grazing on *P. seriata*, the copepodites retained 250 ± 77 and 171 ± 29 ng DA cop^−1^, in the treatments with 12 and 20 copepodites, respectively, corresponding to 2.3 ± 0.7 and 1.6 ± 0.3 ng DA µg C^−1^. The *P. seriata* cells produced on average 8.1 ± 2.5 × 10^−4^ ng DA µg C^−1^.

## 3. Discussion and Conclusions

### 3.1. Calanus Copepodites Grazing on Pseudo-nitzschia

#### 3.1.1. Grazing

In this study we show for the first time that *Calanus* copepodites stages C3 and C4 graze on toxic *Pseudo-nitzschia* and retain the toxin, suggesting that copepodites pose a risk as vectors of DA in arctic marine food. The results suggest that DA did not deter grazing, which may seem as a paradox because the algae induce toxin production in the presence of grazing copepodites. But DA may affect other physical capabilities of the grazer, e.g., the competency to escape predators. This has been seen in the copepods *Oithona similis* and *C. helgolandicus*, which were negatively affected (disoriented or dead) after feeding on a toxic strain of *Alexandrium tamarense* [32]*.* Other potential effects of DA could be a reduction in fecundity and hatching rate of eggs, as has previously been demonstrated for the rotifer *Brachionus plicatilis* [33]. The ingestion and clearance rates did not differ significantly among the treatments or within the treatments, *i.e.*, between the light and dark periods, or on average over the 39 h. The grazing rates were the same in the two experiments and were in the range found in the other studies on *C. finmarchicus* copepodites [34,35]. Our results are in agreement with the results of the majority of previous grazing studies on adult copepods, which have not shown DA to deter grazing by copepods, e.g., no selectivity was observed by [9] for the copepod *Acartia clausi* against the toxic *P. multiseries* compared to the non-toxic *P. delicatissima.* The same was seen for *C. finmarchicus* grazing on toxic *P. multiseries* and non-toxic *P. pungens* [15]*.* The results partly differ from the only study from the same location, where adult females of *C. finmarchicus* and *C. hyperboreus* were temporally affected when fed with toxic *P. seriata* [18], similar to a temporal grazing effect seen on krill [24]. Deterred grazing and/or mortality of copepods have been observed in other toxigenic algal groups, e.g., reduced grazing rates of *C. pacificus* were detected when grazing on toxic dinoflagellates [36,37]. *Acartia tonsa* ingested lower proportion of toxic *Alexandrium minutum* when offered a choice between *A. minutum* and non-toxic alternative prey *Prorocentrum micans* [24], and grazer mortality ranged from 36% to 47% for *Centropages typicus* and *Acartia clausi*, respectively, grazing on toxic *A. minutum* [38]. In the present study, both *Pseudo-nitzschia* species were, to our surprise, found to be toxic, and the copepodites fed continuously on the toxic *P. seriata* as well as on the less toxic *P. obtusa*. Hence, due to the lack of a non-toxic control, we cannot conclude that DA will not have any negative effects on the copepodites, since, e.g., ingestion rates might be higher on a non-toxic species than on the toxic species.

Selectivity for size (grazing differently on *P. seriata*, which is larger than *P. obtusa*) was not observed. This was expected as the copepodites were in the size range of *C. pacificus* and *C. finmarchicus*, and both *P. seriata* and *P. obtusa* are within the size range of prey of these species [18,39]. When culturing clonal strains of pennate diatoms, like *Pseudo-nitzschia*, they inevitably become shorter with time due to restrictions by the silica encasing during cell division—the MacDonald-Pfitzer rule [40]. The large cell size is regained during sexual reproduction. The cell length of the strains of *P. seriata* and *P. obtusa* were therefore small compared to observations of the species in the field. But since the *Pseudo-nitzschia* cells appeared in stepped colonies, which the copepodites apparently did not have difficulties handling, we do not expect that a larger cell size in the field will pose any differences in grazing. The copepodites were similar to the size of an adult *C. finmarchicus*, which has been reported to feed effectively on cells with considerable larger volumes than the cells of the present study [41], it can therefore be expected that the feeding response we see is representative for the feeding on natural populations.

We saw few (six out of 128) dead copepodites in the eight-day induction experiment, and this could to a minor degree be related to a hypothesis of DA not acting as grazing deterrent but as a toxin. As we did not find any effect on grazing as an indication of the copepodites not being well, we do not consider it likely that the six dead copepodites had died because of the DA. Dissolved DA (dDA) has previously been found not to affect the grazing rate but having lethal effects on the copepod *Tigriopus californicus* [42], and dDA has been found to increase mortality of krill [43]. dDA was not measured in the present study, because the same strain had previously been found to leak DA only in very low amounts in exponential growth phase [18], and dDA was thus not considered relevant. Because most copepodites were alive after grazing on monocultures of toxic algae, we assume that neither dDA nor accumulated DA caused copepodite mortality.

The study organisms used here, *Pseudo-nitzschia* and *Calanus*, originate from the same locality, meaning that the *Calanus* population may have a long history of exposure to DA. Local adaptation of copepods to toxins has been studied in *Acartia* spp. grazing on toxic dinoflagellates and has shown that populations with a long history of exposure to toxins show better performance (ingestion rate and egg production) when exposed to toxic algae, in comparison to naïve populations with no previous experience of the toxins [44,45,46,47,48]. If zooplankton populations, e.g., *Calanus* copepodites, are adapted to algal toxins, this may hypothetically, result in a reduced algal cell concentration or shorter duration of a toxic algal bloom due to grazing, compared to presence of naïve zooplankton (*i.e.*, less reduction in grazing). On the other hand, zooplankton populations adapted to algal toxins may accumulate greater quantities of toxins in their bodies, and therefore pose a larger risk for accumulation of toxins in the food web and further increased risk of ASP in humans. Something similar has been observed in the clam *Mya arenaria* where a variation in resistance/sensitivity to paralytic shellfish toxins produced by *Alexandrium tamarense* was found. Clams from areas exposed to the toxins were more resistant to the toxins and accumulated the toxins at higher rates than clams from non-exposed areas, indicating an adaptation to the toxin [49].

The production of fecal pellets confirms that the copepodites were grazing on the algae. The pellet production in this study was 0.3 to 0.4 pellets copepodite^−1^ h^−1^, which is lower than found for *C. finmarchicus* copepodites stages C4 and C5 [35]. This can partly be explained by the accumulation of lipids, which may be higher in stages C3 and C4 than in C5, as suggested in [50], thus illustrating that the younger stages of copepodites are exploiting the ingested food to a higher degree that the older stages. The lipids are essential for the overwintering mechanism and early provision for fueling maturation, gonad development and reproduction in the spring [51,52]. The high degree of food exploitation may further explain the lower weight-specific ingestion rates found when calculations were based on fecal pellets egestion than on clearance of carbon, as the calculations are based on assumptions made on adult copepods [53,54,55,56] (Figure 2c).

#### 3.1.2. Copepodites as Vectors in the Arctic Food Web

This present study is the first conducted on copepodites as potential vectors for DA in the food chain and the results clearly show that the copepodites retain DA. The amount of retained DA, in relation to body weight, ranged from 48 µg DA g DW cop^−1^ when grazing on *P. obtusa*, to 410 µg DA g DW cop^−1^ when grazing on *P. seriata*, for 39 h. This is in range with the amounts in Tammilehto *et al.* [18], where the adult *Calanus* grazed for 12 h on *P. seriata* and the DA retained ranged from 68 µg DA g DW cop^−1^ in *C. glacialis* to 290 µg DA g DW cop^−1^ in *C. finmarchicus*. The body weight of the copepodites was similar to the size of an adult *C. finmarchicus* (~0.11 mg C cop^−1^). The amount of DA retained per copepod, 68 ng DA cop^−1^ when grazing on *P. seriata* for 39 h, is in the range of what has previously been found for adult females; *i.e.*, *C. finmarchicus* was found to accumulate 17 ± 5 ng DA cop^−^^1^ when fed mixed diet of toxic *P. multiseries* and non-toxic *P. pungens*, and 42 ± 4 ng DA cop^−1^ on a diet of *P. multiseries* for 12 h [15]. The results are also similar to the values found in [18], *i.e.*, 55 ± 10 ng DA cop^−1^ for *C. finmarchicus* grazing on *P. seriata* for 12 h. The percentage of ingested DA retained in the copepodites ranged from 6% ± 2% when grazing in *P. obtusa* to 16% ± 4% when grazing on a mixture of both species. This is slightly lower than for adult *Calanus* spp. where values ranged from 37% ± 32% to 48% ± 20% in *C. hyperboreus* and *C. finmarchicus* [18] but in the range of the findings in [15], 12% ± 5% to 34% ± 19%. These relatively high levels of DA retained in copepodites clearly illustrate a potential risk for accumulation of DA in the arctic food web during a toxic bloom of *Pseudo-nitzschia.* The amount of DA retained in *Calanus* copepodites was higher in DA per µg carbon than in the *Pseudo-nitzschia* cells. Furthermore, the levels found in the copepodites were higher than amounts that have been found in krill (<45 μg g DW krill^−1^) [43].

### 3.2. Induction of Domoic Acid Production in Pseudo-nitzschia by Grazing Copepodites

In this study, we saw that DA production was enhanced by 3300%, and reached ~13 pg DA cell^−1^ on day 8, similar to levels was reported by Tammilehto *et al.* [20] who found that toxicity of *P. seriata* (the same strain as used in this experiment) increased significantly in the presence of grazing adult females of *C. finmarchicus* and *C. hyperboreus*. In the present study, DA cell quota was the same in flask B (where the cells were separated from the copepodites) as in flask A (where the cells were in direct contact with the grazers) after day 2, but with a delayed increase in DA cell quota during the first two days, whereas in [20] *P. seriata* was markedly more toxic in flask A than in flask B. This may be explained by the higher densities of grazers used in the present study compared to [20], or that the cues produced by the copepodites may consist of smaller molecules that more easily diffuse over the membrane. Further we can confirm that the cues were not produced by *P. seriata* cells, as the induction was also observed when *P. seriata* cells grew in filtered seawater, where copepodites had been grazing only on *Thalassiosira* sp. The origin of the cues must therefore be the copepodites. Recently, eight cueing compounds, copepodamides, were isolated and identified from *Centropages typicus*, *Pseudocalanus sp.* and *Calanus finmarchicus*, and found to be responsible for toxin induction in *Alexandrium minutum* [57]. The copepodamides differed among copepodites, and toxin production in *A. minutum* was shown to be compound specific. Species-specific elevation of toxin production has previously been reported for dinoflagellates [32,38] and for our strain of *P. seriata*.

Triggers for induced DA production have previously been found to be environmental factors, e.g., nutrient levels, reviewed in [11], and pH [23,58]. *Pseudo-nitzschia* has been found to increase toxin production when stressed by depletion of silicate or phosphate [21], and when ammonium or nitrate levels are replete [22,59]. The growth medium in this study was 1/10 of L-medium with additional ammonium spiked into filtered seawater from the locality (see in detail in Material and Methods) in order to supply the cells with enough nutrients for growth and DA production and to avoid artifacts from extreme nutrient conditions. The measured initial nutrient levels were, however, low for both silicate and phosphate, due to the low nutrient levels in the seawater used. Levels of silicate decreased significantly in treatments and in the controls, but the increase in the toxicity of *P. seriata* was only seen in the treatments with copepodites (Table 2 and Supplementary Table S1) and not in the controls, excluding silicate depletion as trigger for DA production. At the end of the experiment, silicate levels were below 1 µM in the control, which is the level previously considered as depleting, and where *Pseudo-nitzschia* spp. have previously been reported to induce DA production [21], however, in this study, depletion of silicate did not enhance DA production. Silicate was significantly higher in the treatments than in the controls at the end of the experiment, but to our knowledge, silicate depletion has never been shown to inhibit DA production in *Pseudo-nitzschia*. Phosphate concentrations decreased in the control but remained the same in the treatments, except in flask A with 20 copepodites, where phosphate increased significantly. In addition, phosphate was never below the depleting 1 µM [21], and we can therefore exclude phosphate depletion as DA-inducing factor. The results show higher levels of silicate, phosphate and nitrate in the treatments with copepodites compared to the controls, and our assumption is that they partly derive from the intracellular content of inorganic nutrients released into the medium when the grazers crush the silicate frustules during grazing. Copepods may leak ammonium and high levels (>200 μM) can lead to increased cellular DA quota in *Pseudo-nitzschia* [22]. Ammonium concentrations were the same in the control and the treatment, except for higher levels in one treatment (flask B, 20 copepodites) (Table 2), and the levels of ammonium were not in the range shown to cause increased DA-production [22]. Therefore, levels of ammonium cannot explain the induced DA production. High nitrate levels may also trigger DA production [22,59]. Nitrate concentrations were significantly higher in the treatments than in the control at the end of the experiment in the induction experiments. The source of the nitrate most likely originates from the addition of cells to the A-flasks on day 3. The input of cells may have resulted in addition of nutrients at higher levels than the 1/10 medium added to the controls, as the addition was based on a mixture of 1/10 medium of cells from a full medium culture. Another possibility is, as mentioned above, the nitrate might originate from the *Pseudo-nitzschia* cells when crushed during grazing. Further, the nitrate might originate from the copepodites via nitrification occurring on the carapace of the animals, a phenomenon that is not uncommon among aquatic invertebrates [60]. The ammonium excretions of the copepodites would be a source of ammonium for ammonia-oxidizing bacteria [61]. To support this hypothesis an increase in nitrogen has been detected in incubation of starving *C. hyperboreus* from the same locality (Peter Stief, personal communication) [62]. It should be pointed out that the levels of nitrate at the start of both the 39 h induction experiment and the grazing experiment were in range with the end concentration of the eight-day induction experiment, and as the latter experiment controls and treatment end concentrations did not differ in spite of different DA levels, we thus exclude nitrate as a trigger.

Studies conducted on natural population have demonstrated that low silicate Si(OH)_4_:PO_4_ and Si(OH)_4_:NO_3_ ratios correlated with high DA-production, suggesting silicate limitation causing toxin production [63]. However, at the same locality, [64] reported correlations between low ratios of Si(OH)_4_:PO_4_^−3^ and (NO_3_^−^ + NO_2_^2−^):PO_4_^3−^ correlated with the enhancement in DA production, but low Si(OH)_4_:(NO_3_^−^ + NO_2_^2−^) ratios did not. These studies are partly in agreement with the results of laboratory results showing that silica stress increases toxin production in *Pseudo-nitzschia*. Other studies have, however, not detected any correlation with ambient concentrations of the nutrients and DA production [65,66]. Further, DA production in natural population of *Pseudo-nitzschia* has been measured when the levels of nutrient where replete [67]. In the present study, the nutrient levels of the local seawater were not available prior to the experiments and not within the Redfield ratio. In both the treatment and the control, the nutrient levels were closer to the ratio suggested to elicit DA production; nonetheless, DA was not induced in the controls. Our findings are therefore not in agreement with the previous in laboratory experiments, and the divergence between the field studies further clearly demonstrates that there is a lack of a strong relationship between the level of nutrient levels and DA production in the field. Studies exploring the interaction of factors inducing DA production are needed to improve our understanding of the factors inducing DA production in the field.

The cellular toxin levels in the present study were higher than previously seen in some laboratory experiments with *P. seriata*, where e.g., phosphate as the depleting factor resulted in a maximum of 2.9 pg DA cell^−1^, but were comparable to levels under silicate depletion were the cells produced max 14.7 pg DA cell^−1^ [22]. The levels of DA induced during exponential growth phase are within the range seen in *P. seriata* in the field, e.g. a concentration of up to 21 pg DA cell^−1^ was reported in [68] in Danish waters. Similar concentrations have been measured from Danish, Scottish and Canadian strains, which yielded DA concentrations of up to 33.6 pg cell^−1^, 14.7 pg cell^−1^ and 7 pg cell^−1^, respectively [69,70]. The levels of DA are further in the range found in the field for other *Pseudo-nitzschia* species e.g., 0.1–78 pg cell^−1^ in *P. australis* [71], 7–75 cell^−1^ in field samples comprising mainly *P. australis* [72] and 0–117 pg DA cell^−^^1^ in samples containing a mixture of *Pseudo-nitzschia* species [64].

pH levels in the present study ranged between 8.1 and 8.2 and no significant differences were found between the start and end of the experiment or among treatments and controls. Therefore, pH as well as nutrient concentrations can be excluded as a cause for the inducing increased DA-production in *P. seriata* and *P. obtusa*.

### 3.3. Toxin Production in the Previously Non-Toxic P. obtusa

Our initial intention was to use *P. obtusa* as a non-toxic control strain to explore the effect of toxic diet on the copepodites. *P. obtusa* has previously been tested negative for DA during different growth phases, as well as at pH levels from 8.0–9.1 [58,73]. Initial tests showed that *P. obtusa* did not produce DA at present detection levels, but surprisingly it was revealed to be toxigenic when induced by copepodites. Whether *P. obtusa* simply produced DA at levels below the detection limit and toxin production was enhanced by the induction, or whether the toxin production was completely shut down before the induction and the production was turned on by the induction, cannot be determined in the present study and needs further attention, as well as the possibility that other *Pseudo-nitzschia* species are toxin producing when exposed to grazers.

### 3.4. Overall Conclusion

*Calanus* copepodites stages C3 and C4 grazed on toxic *Pseudo-nitzschia* and retained high levels of the toxin, suggesting that the copepodites are able to tolerate DA and act as vectors of DA to higher trophic levels, thus posing a threat to the arctic marine food web and the humans exploiting it. The presence of grazing copepodites induced DA production in both *Pseudo-nitzschia* species, suggesting that production of DA by *Pseudo-nitzschia* may be a defense mechanism against grazing, however in this study no effect were found on the grazing rate. An induced response was also elicited when the copepodites had previously been grazing on another diatom species, illustrating that the cues do not originate from *Pseudo-nitzschia* cells. The results from both experiments show that the induced response in production of DA is not caused by changes in nutrient levels or pH, but suggest that the water borne cues originate from the copepodites. Finally, the present study is the first report of *P. obtusa* being a toxin-producing *Pseudo-nitzschia* species, the 15th known DA producer of this genus.Copepods, including copepodites, constitute an imperative link, transferring energy from primary producers to higher trophic levels, in marine food webs and consequences of an increase of 3300% increase in DA production due to grazing of copepodites may be profound for the marine food web, with further consequences for human health and economy.

## 4. Materials and Methods

### 4.1. Study Organisms

#### 4.1.1. Zooplankton

Mesozooplankton was collected from Disko Bay (69°14′ N, 53°23′ W), West Greenland, in June 2013, from the upper 100 m using a WP-2 net (200 µm). *Calanus* in stages C3 and C4 were identified by the number of urosome somites and picked individually in petri dishes placed on ice blocks using stereo microscopes (Nikon SMZ-1B and Leica Wild M3b). The copepodites were kept in the dark at 4 °C for a maximum of 2 weeks in 0.22 µm filtered seawater and fed with *Thalassiosira* sp. Prior to the experiments, the copepodites were starved for 24 h. Carbon content of the copepodites (C) was calculated from a length/weight regression using the equation given for post bloom period; C = 0.0044 × PL^3.57^, where PL is prosome length (Table 3). Dry weight was calculated assuming the carbon weight to dry mass ratio to be 0.60 [53].

**Table 3:** Size and carbon content of the study organisms. Cell length and width (µm), cell volume (µm^3^), and carbon content (pg C cell^−1^) for *Pseudo-nitzschia obtusa* and *P. seriata*. Length promosome (mm) and carbon weight of the copepodites, n is the number of cells/individuals measured and values are given in mean ± SD.

***Pseudo-nitzschia***	**Cell Length (µm)**	**Cell Width (µm)**	**Cell Volume (µm^3^)**	**Carbon Content (pg C cell^−1^)**
*P. obtusa* *n =* 23	33.5 ± 3.2	4.2 ± 0.5	489 ± 149	57.3
*P. seriata* *n =* 23	49.0 ± 3.4	5.4 ± 0.8	1163 ± 383	137.1
***Calanus***	**Prosome length (mm)**			**Carbon content per individual (mg cop^−1^)**
*n =* 21	2.5 ± 0.5			0.11 ± 0.1

For calculations, phytoplankton growth rate in the controls was calculated using an exponential model and changes in phytoplankton concentrations in the grazing were calculated after [39]. Ingestion rate was calculated as number of food items consumed by each grazer and weight-specific ingestion was estimated by multiplying number of *Pseudo-nitzschia* cells ingested by the cellular carbon content and dividing it by the average carbon body weight of copepodites. For the equations in detail see [18].

At the end of the experiment, fecal pellets were counted and the size estimated. The width of >20 fecal pellets per treatment and the length >30 fecal pellets per replicate were measured, and only pellets that were at least 3× longer than wide were included. Fecal pellet volume was calculated assuming a cylinder shape and the carbon content was estimated by applying a volume to a carbon conversion factor of 0.043 pg C μm^−3^ [54]. Specific fecal pellet production (SPP) was calculated according to [54] using number of fecal pellets, the average volume of fecal pellets and the pellet volume to carbon conversion factor. Weight-specific ingestion was calculated from fecal pellet production, *i.e.*, egestion and assimilation efficiency followin*g* [55], and the assimilation efficiency was assumed to be 0.65 after [56]. All calculations were conducted following the equations as they appear in [18].

#### 4.1.2. Phytoplankton

Strains of *P. seriata* (strain P5G3) and *P. obtusa* (strain L4B4) were isolated into clonal cultures from Disko Bay (69°14′ N, 53°23′ W), in April 2010 and in April 2012, respectively, by isolating single cells or chains into micro well plates, and later inoculating the cultures into tissue culture flasks. Strain P5G3 was known to be a toxic strain of *P. seriata*, whereas *P. obtusa* was previously only known as a non-toxic species [73]. Strain P5G3 is the same strain as used in the previous experiments on *Calanus* and *Pseudo-nitzschia* in Disko Bay by Tammilehto *et al.* [18,20]. Prior to the experiments, both species were grown in batch cultures at 4 °C in L1-medium [74] under a light intensity of 85 µmol photons m^−2^s^−1^, at a 18:6 light:dark cycle using cool white fluorescent bulbs. Carbon cell quota was calculated using measured carbon content of *P. multiseries* (0.12 pg C µm^−3^) following [19] and relating this to the cell volume of *P. seriata* and *P. obtusa*. The cell volume of *Pseudo-nitzschia* spp. was calculated following [23]: volume = (0.6 × *L* × *W*^2^) + (0.4 × 0.5 × *L* × *W*^2^), where *L* is the cell length and *W* is the cell width (Table 3).

### 4.2. Experiment Preparation, Nutrient and DA Analyses

Actively swimming copepodites were sorted out in culture flasks filled with 0.22 µm filtered seawater. Triplicate subsamples of copepodites were taken for DA analysis, where each replicate contained the same number of specimens as used in the experimental flasks. The copepodites were rinsed and filtered onto GF/F filters using gentle vacuum, and frozen at −20 °C until toxin analysis.

Targeted initial diatom cell concentrations were prepared by diluting the cultures of *P. seriata* and *P. obtusa* with an appropriate volume of 1/10 L1-medium. The initial cell concentrations provided copepodites with saturating carbon supply (>400 µg C L^−1^) [75]. To avoid artifacts from extreme nutrient concentrations, 1/10 L1 medium was used, based on 0.22 µm filtered seawater with a salinity of 36. Additional ammonium chloride (0.1 mL of 500 mM NH_4_Cl L^−1^) was added to the medium to avoid large differences in ammonium concentrations among treatments due to ammonium excreted by copepods [76]. Local seawater was used for the experiments, and we were unfortunately not able to measure nutrient levels before performing the experiment, and therefore the initial levels of silicate and phosphate were lower than anticipated. pH was measured using a WTW pH 3110 pH-meter with a SenTix 41 electrode (a sensor detection limit of 0.01; two point calibration) (WTW, Xylem), and triplicate subsamples of 50 mL for inorganic nutrient composition were taken. For measuring DA cell quota, triplicate subsamples of 200 mL *P. seriata* and *P. obtusa* were taken from the final culture for the experiments, and filtered onto GF/F filters. Nutrient samples and samples for DA analysis were stored at −20 °C until analysis. The nutrients were analyzed at the Institute for Bioscience, Aarhus University in Denmark on a flow injection auto-analyzer, following [77]. DA analyses were conducted by liquid chromatography coupled with tandem mass spectrometry as described in details in [18]. Detection limit for DA was 1 ng sample^−1^. All experiments were run at a temperature of 4 °C, and a light intensity of 100 µmol photons m^−2^s^−1^ and 12:12 light:dark cycle using cool white fluorescent bulbs. The experimental flasks were mounted on a plankton wheel with speed 1.3 rpm.

### 4.3. Grazing Experiment

The copepodites were fed with cultures of toxic *P. seriata*, non-toxic *P. obtusa*, and a mixture of both species. Each of the three grazing experimental treatments were carried out in 720 mL polystyrene flasks (Sarstedt) in four replicates with 12 copepodites per flask and run in parallel with controls in 3 replicates for 39 h. The nominal initial cell concentration was 4000 cells mL^−1^ of *P. seriata* and 5000 cells mL^−1^ of *P. obtusa*, and 2000 cells mL^−1^ of *P. seriata* plus 5000 cells mL^−1^ of *P. obtusa* in the mixed culture for both treatments and controls (Table 3).

At the beginning and at the end of the experiment, samples for nutrients and DA as well as pH measurements were done as described in Section 4.4. Subsamples for enumerating cell concentrations were taken every 3 h, and the volume removed was replaced with 0.22 µm filtered seawater. At the end of the experiment (39 h), the copepodites and fecal pellets were collected as described in Section 4.4.

### 4.4. Induction Experiment

Induction experiment was carried out in specially designed incubators, made of two 720 mL polystyrene tissue culture flasks connected via two apertures (4.5 cm in diameter) with a 2 µm polycarbonate membrane, see details in Tammilehto *et al.* [20]. The transmission of food dye through the membrane was measured on a synergy scanner (Biotek), and the diffusion equilibrium was ~30% after two days, matching the diffusion over a similar membrane in a set up made by Tang *et al.* [78]. The membrane allowed water exchange between the flasks but the plankton remained in their initial flasks, named A and B for the two sides. Cells of *P. seriata* and copepodites in concentrations of either 12 or 20 individuals were inoculated into flask A, and only *P. seriata* into flask B. The initial cell concentration of *P. seriata* was 3000 cells mL^−1^. The experiment was run in 4 replicates and a control in 3 replicates (using single flasks, 720 mL) for eight days.

Subsamples for cell counts (2 mL; fixed with 2% (final concentration) acidic Lugol’s solution) and DA analyses (200 mL) were taken on days 0, 2, 5 and 8. The volume sampled was replaced with 0.22 µm filtered seawater. On day 3, 168 mL of *P. seriata* culture of approximately 7000 cells mL^−1^ was added into flask A to supplement for a substantial amount of cells grazed by the copepodites and to ensure the copepodites a sufficient amount of cells for grazing. Similarly, 168 mL of 1/10 L1 medium was added to the controls and flask B. New controls in three replicates were subsequently established for measuring growth rate of the cells that had been added to the treatments. The cells originated from a culture based on a mixture of 1/10 L1 medium and cells grown in full medium. At the end of the experiment (day 8), pH was measured and subsamples for cell enumeration, nutrients and DA analyses were taken as described above. The copepodites were collected by sieving the total content through a 200 µm sieve to remove phytoplankton and fecal pellets. The copepodites were rinsed with 0.22 µm filtered seawater, and individually transferred to a GF/F filter and stored at −20 °C.

### 4.5. Induction Experiment in Filtrate Water

Filtrate water was prepared by placing >70 actively swimming copepodites into 0.22 µm (~6 L) filtered seawater with a unialgal culture of *Thalassiosira* sp. for 24 h. The copepodites and the fecal pellets were removed by using a 50 µm sieve and the cells of *Thalassiosira* sp. removed by filtering through a 0.22 µm filter. Experimental flasks with an initial cell concentration of 4000 cells mL^−1^ of *P. seriata*, was prepared using this filtrated water using 720 mL polystyrene flasks. The experiment was conducted in four replicates and three controls and run on the plankton wheel simultaneously for 39 h with the grazing experiment (Section 4.3).

### 4.6. Microscopy

For enumeration of the algal cells, a Sedgewick-Rafter chamber and an inverted light microscope (Olympus CKX31 at a 100× magnification). A minimum of 400 cells were counted. Fecal pellets were counted using a stereo microscope (Reichter at 16× magnification) and width × length of the fecal pellets was measured using 40× magnification. For measurements of *Calanus* and *Pseudo-nitzschia* see Section 4.1.1 and Section 4.1.2.

### 4.7. Statistical Analyses

Normal distribution of data was tested using a Shapiro-Wilk test and homogeneity of variances applying Levene’s test. The differences over time within each treatment were tested by repeated-measures ANOVA (RM ANOVA) and *post hoc* analysis tested using Holms-Sidak adjustments. Differences between means were tested using one-way ANOVA, a *t*-test or a paired *t*-test. If the data set did not pass the assumption of normal distribution, a Mann-Whitney U-test or a Kruskall-Wallis test were used. For the nutrients measurements in Table 2 and Supplementary Table S1, the statistical analyses were conducted with paired *t*-test between the start and end, and *t-*test between the control end and the treatments end. Results given as * = significant difference from control start an ** for significant difference from the control end. The level of significance used was 0.05.

## Supplementary Files

Supplementary File 1
