# Supplementary material for: Dangerous Relations in the Arctic Marine Food Web: Interactions between Toxin Producing Pseudo-nitzschia Diatoms and Calanus Copepodites
Source: Mar Drugs. 2015 Jun 16;13(6):3809–35. doi: 10.3390/md13063809 (PMC4483658; doi:10.3390/md13063809)
Supplement: Supplementary File 1 [file marinedrugs-13-03809-s001.pdf]

Supplementary Information

Table S1. Nutrient concentrations at start and end of the grazing experiments. Number of replicates was n = 3 except for the nitrate control start with n = 2. Values are given as mean ± SD. Results are given as * = significant difference from control start $P < 0.05$ and ** = significant difference from the control end.

Time (h)	Treatment	$SiOH_4$ (µmol L^{-1})	PO_4^{3-} (µmol L^{-1})	NH_4^+ (µmol L^{-1})	NO_3^- (µmol L^{-1})	pH
0	*P. seriata*	6.7 ± 0.6	7.0 ± 0.2	38.8 ± 1.7	164.8 ± 12.5	8.08 ± 0.00
0	*P. obtusa*	4.3 ± 1.3	8.2 ± 0.9	35.5 ± 4.4	207.7 ± 32.8	8.08 ± 0.01
0	*P. seriata* + *P. obtusa*	5.6 ± 0.1	8.1 ± 0.2	39.3 ± 1.1	203.7 ± 4.1	8.09 ± 0.00
39	*P. seriata*	3.6 ± 0.3 *	7.4 ± 0.1 *	39.9 ± 1.0	177.7 ± 1.0	8.11 ± 0.00
39	*P. obtusa*	1.4 ± 0.3 *	8.6 ± 0.0	36.9 ± 0.7	229.1 ± 1.5	8.18 ± 0.00
39	Mix of *P. s* and *P. o*	2.4 ± 0.3 *	7.7 ± 0.2	37.9 ± 0.2	194.3 ± 4.2	8.16 ± 0.01
39	*P. seriata* + copepodites	4.6 ± 0.3	7.7 ± 0.3 *	41.9 ± 1.0 *	176.4 ± 3.8	8.10 ± 0.01
39	*P. obtusa* + copepodites	2.4 ± 0.5 **	8.5± 0.2	39.7 ± 0.6 **	225.2 ± 3.7	8.17 ± 0.01
39	Mix of *P. s.* and *P. o.* + copepodites	3.6 ± 1.1	8.6 ± 0.7	40.7 ± 0.2	201.8 ± 4.9	8.14

Table S2. Domoic acid (DA) cell quota of *Pseudo-nitzschia seriata* (pg DA $cell^{-1}$) in induction experiments on days 0–8 (mean ± SD) in control, flask A with cells and copepodites, and flask B with cells separated from copepodites with a membrane. n = number of copepodites.

Time (day)	Control (pg DA $Cell^{-1}$)	Flask A n = 12 (pg DA $Cell^{-1}$)	Flask B n = 12 (pg DA $Cell^{-1}$)	Flask A n = 20 (pg DA $Cell^{-1}$)	Flask B n = 20 (pg DA $Cell^{-1}$)
0	0.4 ± 0.1				
2	0.3 ± 0.1	4.3 ± 0.6	0.6 ± 0.2	3.3 ± 0.9	0.7 ± 3.0
5	0.3 ± 0.1	6.3 ± 1.0	5.0 ± 1.8	5.5 ± 1.5	6.5 ± 1.8
8	0.4 ± 0.0	13.3 ± 4.9	9.2 ± 2.8	9.5 ± 3.0	12.4 ± 1.9

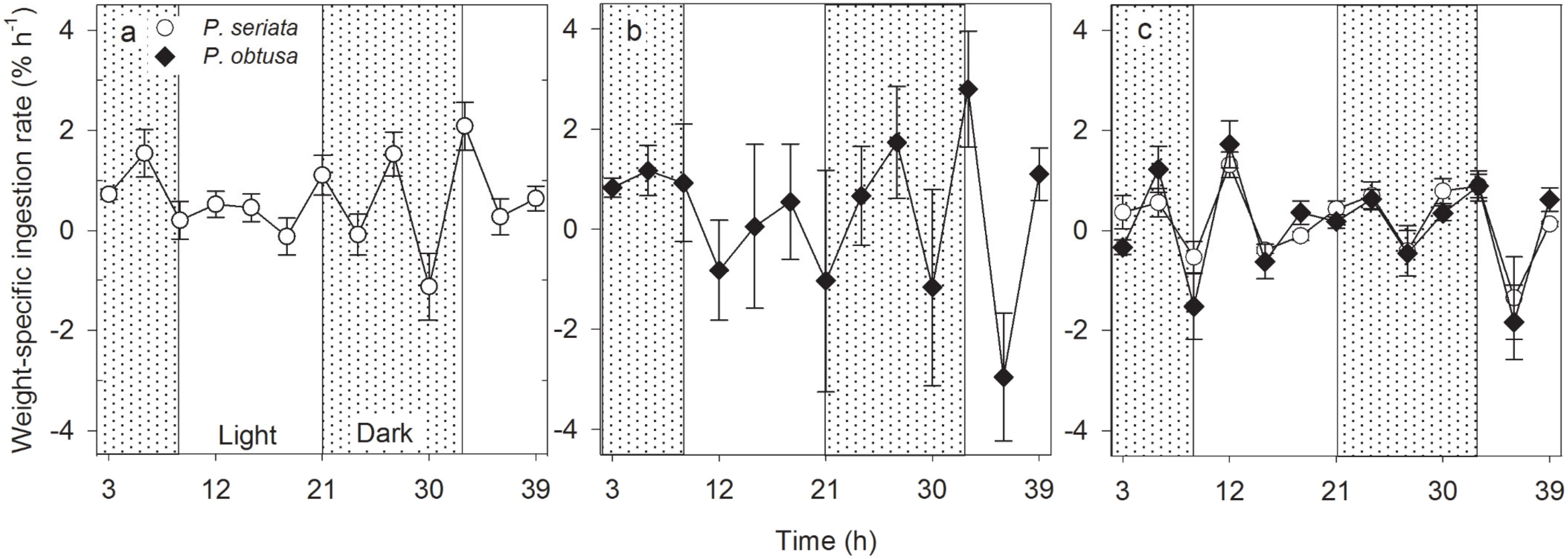

Figure S1. Grazing experiment. Weight-specific ingestion rates (percentage per hour, mean ± SD) for grazing on (**a**) *Pseudo-nitzschia seriata* (**b**) *P. obtusa* (**c**) and a mix of both species, during a 39 h-period. A 12:12 light:dark period was used, and the periods are marked as light or dark time periods.

© 2015 by the authors; licensee MDPI, Basel, Switzerland. This article is an open access article distributed under the terms and conditions of the Creative Commons Attribution license (http://creativecommons.org/licenses/by/4.0/).
